# Selective Detection of Misfolded Tau From Postmortem Alzheimer’s Disease Brains

**DOI:** 10.3389/fnagi.2022.945875

**Published:** 2022-07-20

**Authors:** Ling Wu, Zerui Wang, Shradha Lad, Nailya Gilyazova, Darren T. Dougharty, Madeleine Marcus, Frances Henderson, W. Keith Ray, Sandra Siedlak, Jianyong Li, Richard F. Helm, Xiongwei Zhu, George S. Bloom, Shih-Hsiu J. Wang, Wen-Quan Zou, Bin Xu

**Affiliations:** ^1^Department of Pharmaceutical Sciences, Biomanufacturing Research Institute and Technology Enterprise (BRITE), North Carolina Central University, Durham, NC, United States; ^2^Department of Biochemistry, Virginia Polytechnic Institute and State University, Blacksburg, VA, United States; ^3^Department of Pathology, Case Western Reserve University, Cleveland, OH, United States; ^4^Departments of Biology, Cell Biology, and Neuroscience, University of Virginia, Charlottesville, VA, United States; ^5^Department of Pathology and Neurology, Duke University Medical Center, Durham, NC, United States; ^6^School of Neuroscience, Virginia Polytechnic Institute and State University, Blacksburg, VA, United States

**Keywords:** selective detection, RT-QuIC, Alzheimer’s disease, protein aggregation, tau isoforms

## Abstract

Tau aggregates are present in multiple neurodegenerative diseases known as “tauopathies,” including Alzheimer’s disease, Pick’s disease, progressive supranuclear palsy, and corticobasal degeneration. Such misfolded tau aggregates are therefore potential sources for selective detection and biomarker discovery. Six human tau isoforms present in brain tissues and both 3R and 4R isoforms have been observed in the neuronal inclusions. To develop selective markers for AD and related rare tauopathies, we first used an engineered tau protein fragment 4RCF as the substrate for ultrasensitive real-time quaking-induced conversion analyses (RT-QuIC). We showed that misfolded tau from diseased AD and other tauopathy brains were able to seed recombinant 4RCF substrate. We further expanded to use six individual recombinant tau isoforms as substrates to amplify misfolded tau seeds from AD brains. We demonstrated, for the first time to our knowledge, that misfolded tau from the postmortem AD brain tissues was able to specifically seed all six full-length human tau isoforms. Our results demonstrated that RT-QuIC analysis can discriminate AD and other tauopathies from non-AD normal controls. We further uncovered that 3R-tau isoforms displayed significantly faster aggregation kinetics than their 4R-tau counterparts under conditions of both no seeding and seeding with AD brain homogenates. In summary, our work offers potential new avenues of misfolded tau detection as potential biomarkers for diagnosis of AD and related tauopathies and provides new insights into isoform-specific human tau aggregation.

## Introduction

**FIGURE 1:**
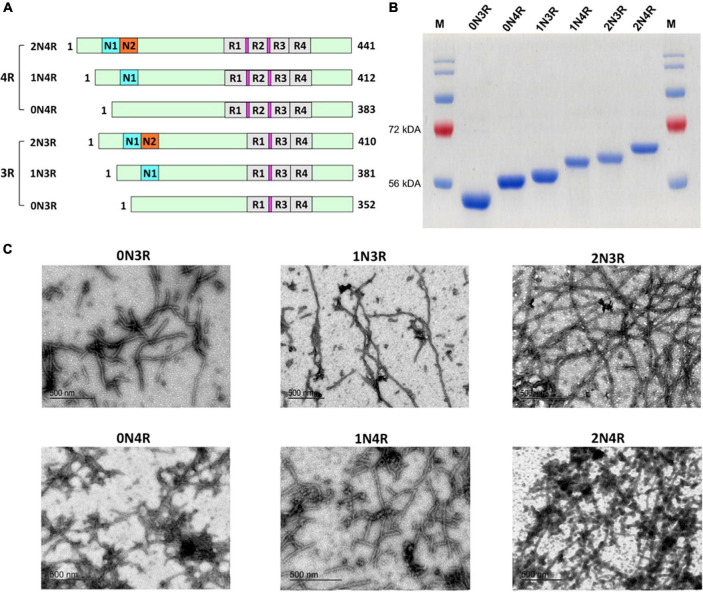
Human tau isoforms. **(A)** Schematic illustration of the alternative spliced six isoforms of human CNS tau. Amino-terminal insert domains (N1 and N2) and carboxy-terminal repeat domains (R1-R4) are shown. Amino acid residue numbers for each isoform are listed. Locations for the hexapeptide sequences (VQIINK in R2 and VQIVYK in R3) are highlighted in magenta. **(B)** SDS-PAGE analysis demonstrated six recombinant human tau isoforms purified to homogeneity. **(C)** TEM images of mature filaments of all six human tau isoform are shown. 30 μM of each tau isoform was prepared in 20 mM Tris pH 7.4 with 0.06 mg/ml heparin and incubated at 37°C for 12 days. Mature tau isoform fibrils were visualized after staining with uranyl acetate.

Alzheimer’s disease (AD) is characterized by the accumulation in the brain of two types of abnormal structures in the brain, extracellular Aβ amyloid plaques and intraneuronal tau neurofibrillary tangles (Braak and Braak, 1991; Selkoe, 2001). Until recently, plaques and tangles were thought not only to represent molecular hallmarks of AD, but also to cause the synaptic dysfunction and neuronal death that lead to the memory and cognitive deficits characteristic of AD patients (Bloom, 2014; Spires-Jones and Hyman, 2014). Several lines of evidence have suggested that pathological changes in tangles correlate better with neuronal dysfunction than Aβ deposits (Wilcock and Esiri, 1982; Nelson et al., 2012). Moreover, a close relationship between tau aggregates and neuronal loss is well established in hippocampus and cerebral cortex tissues (Goedert and Spillantini, 2017). Tau aggregates are present not only in AD brains, but also in multiple neurodegenerative diseases known as “tauopathies” (Arai et al., 2001; Lee et al., 2001), including Pick’s disease (PiD), progressive supranuclear palsy (PSP), and corticobasal degeneration (CBD). Six tau isoforms are expressed in adult human brain, produced by alternative mRNA splicing of transcripts from *MAPT* gene. These isoforms contain either three or four microtubule-binding repeats (3R or 4R tau) and 0-2 N-terminal inserts (0N, 1N, or 2N tau) (Figure 1A) (Goedert et al., 1989). Significantly, isoform composition and morphology of tau filaments can differ between tauopathies, suggesting the existence of distinct misfolded tau strains, molecular heterogeneity and complexity of these tauopathy diseases (Kaufman et al., 2016; Dujardin et al., 2018; Vaquer-Alicea et al., 2021). In AD, both 3R and 4R isoforms make up the neuronal inclusions, whereas in PiD, 3R isoforms predominate in the neuronal deposits. The assembly of 4R tau into filaments is a characteristic of PSP and CBD. Transmission of the AD pathology and related tauopathies is not fully understood, but is believed to be through “prion-like seeding” mechanisms that ultimately yield intercellular spreading of toxic tau aggregates (Guo and Lee, 2011, Guo et al., 2016; Swanson et al., 2017; Gibbons et al., 2019; Vaquer-Alicea and Diamond, 2019; Goedert, 2020).

**FIGURE 2:**
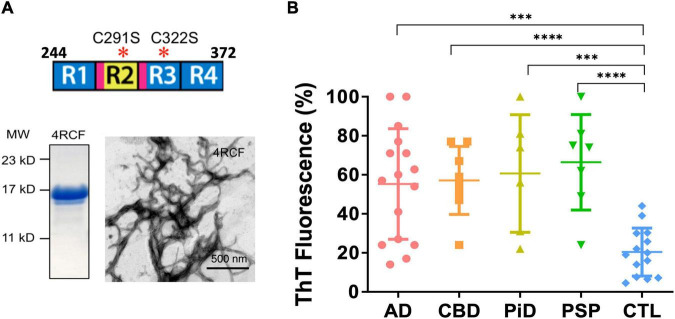
Recombinant truncated tau protein 4RCF and scattered plot of tau-aggregation seeding activity (tau-ASA) of brain tissues from tauopathies. **(A)** Schematic representation of truncated tau protein (upper panel; four repeating segments of 2N4R only, with two cysteine to serine mutations – cysteine free, therefore its name 4RCF; it covers the four-repeating segment of tau by spanning the amino acid sequence from 244 to 372 of the longest isoform 2N4R tau). Locations for the hexapeptide sequences (VQIINK in R2 and VQIVYK in R3) are highlighted in magenta. SDS-PAGE of purified recombinant 4RCF and corresponding 4RCF fibrils transmission electron microscopy (TEM) image are shown in the lower panels. **(B)** Tau-ASA is represented by the percentage of tau ThT fluorescence at the endpoint of brain tissues after real-time quaking-induced conversion analyses (RT-QuIC) assay. Corresponding time-dependent ThT fluorescence kinetic curves are shown in Supplementary Figure 4. The brain tissues from Alzheimer’s disease (AD) (*n* = 16), CBD (*n* = 8), PiD (*n* = 6), PSP (*n* = 7) and control (CTL, *n* = 14) were examined by RT-QuIC assay in the presence of the 4RCF tau substrate. AD: 55.3 ± 28.4 (average ± SD); CBD: 57.1 ± 17.5; PiD: 60.7 ± 30.1; PSP: 66.4 ± 24.5 and CTL: 21.0 ± 12.1. ^****^p < 0.0001; ^***^p < 0.0004.

Traditionally, definitive diagnosis of AD relies on postmortem neuropathological examination and confirmation of the presence of both neurofibrillary tangles and Aβ plaques. More recently, clinical diagnosis of AD and AD-related dementia (ADRD) is supported by imaging biomarkers such as positron emission tomography which is relatively expensive for patients, or by CSF biomarkers such as Aβ42, Aβ42/40 ratio, phosphorylated tau, and total tau which involves an invasive spinal tap procedure. Identifying biomarkers for the development of non-invasive or minimally invasive and inexpensive testing across AD and ADRD is an urgent and unmet need. Many protein misfolding diseases, such as AD (Chiti and Dobson, 2017), Parkinson’s disease (Lee and Trojanowski, 2006), prion disease (Prusiner, 2013), and type 2 diabetes (Westermark et al., 2011; Wu et al., 2021), involve analogous pathological accumulation of disease-specific amyloidogenic protein in the form of self-seeding filaments or sub-filamentous deposits. An ultrasensitive detection method of misfolded proteins, named real-time quaking-induced conversion (RT-QuIC), originated from prion disease detection (Moda et al., 2014; Orru et al., 2017; Wang et al., 2019), gained significant application for *in vitro* amplification in a number of neurodegenerative diseases, such as Aβ oligomers in AD (Salvadores et al., 2014) and α-synuclein in Parkinson’s disease (Fairfoul et al., 2016; Shahnawaz et al., 2017; Groveman et al., 2018; Wang et al., 2020). Using engineered short fragments of tau (K19 or K12) or a mixture of tau fragments (K19 + τ306, K18 + K19) as substrates, specific detection of the prion-like tau seeding activities by RT-QuIC assays had been reported with autopsy brain tissues of AD, Pick’s disease, and chronic traumatic encephalopathy (Saijo et al., 2017; Kraus et al., 2019; Metrick et al., 2020). However, whether other tau fragments (such as K18-mimicking 4RCF alone), can serve as effective substrates to amplify the misfolded tau seeds relevant to the pathogenesis of AD and related tauopathies are not well studied. 4RCF tau is an engineered, truncated tau fragment spanning 2N4R amino acid sequence 244-372 (four repeat segments of 2N4R; Figure 2A), the essential segments that contribute to 4R tau aggregation. Of particular interest and importance, full-length wild-type human tau isoforms, the *bona fide* forms of tau protein in the brain, have not been studied as effective seeding substrates for AD and related tauopathies. Given the complexity of molecular pathology of AD and related tauopathies, a diverse set of tau substrate constructs will provide a versatile toolbox for selective detection of AD and other tauopathies in diagnosis, differentiation, and disease course prognostication using ultrasensitive detection technologies such as RT-QuIC and protein misfolding cyclic amplification (PMCA) (Saá et al., 2005; Shahnawaz et al., 2020).

In this study, we demonstrated that prion-like seeding activities of misfolded tau from post-mortem AD brains with not only a novel engineered tau fragment 4RCF, but also all six full-length 3R-tau and 4R-tau isoforms as effective substrates. The results suggested that selective use of various recombinant human tau isoforms or fragments may be critical in developing RT-QuIC-based diagnosing, characterizing, and predicting consequences of AD and non-AD tauopathies. Our kinetic measurements also revealed isoform-specific aggregation properties that 3R tau isoforms aggregated significantly faster than their 4R counterparts, which may have physiological or pathological significance as we gain better understanding of poorly understood functions of six tau isoforms in human brains.

## Materials and Methods

### Plasmid Constructs

Expression vectors for his-tagged versions of all six wild-type human tau isoforms were kindly provided by the late Dr. Lester “Skip” Binder and Dr. Nicolas Kanaan of Michigan State University. 4RCF construct (four microtubule-binding repeats and cysteine-free construct containing C291S and C322S mutations) was first PCR-amplified of 4R repeats sequence from 2N4R tau plasmid and cloned into the same expression vector using *Nde*I and *Xho*I restriction sites, followed by site-directed mutagenesis at Cys291 and Cys322 sites using QuikChange Site-directed mutagenesis kit (Agilent, Santa Clara, CA). All constructs were designed with a his_6_-tag at their carboxy-termini to facilitate protein purification and were verified by DNA sequencing.

### Brain Tissue Collection, Handling and Analysis

The autopsy brain samples were collected and diagnosed neuropathologically at Case Western Reserve University Alzheimer’s Disease Research Center and Case Human Tissue Procurement Facility. Diagnostic guidelines follow the National Institute on Aging-Alzheimer’s Association workgroups (McKhann et al., 2011). All protocols currently in place were to support and facilitate the research in the context of IRB and HIPAA regulations, by articulating and implementing criteria allowing the acquisition, storage and distribution for research use of such data and specimens with or without linkage to personal health identifiers. Postmortem brain tissues for research usage was approved by the Case Western Reserve University’s Institutional Review Board (IRB) with informed consent from patients or their families. Additional postmortem control and rare tauopathy brain samples were collected from the Bryan Brain Bank and Biorepository of the Duke University/University of North Carolina Alzheimer’s Disease Research Center (Duke/UNC ADRC). All participants were enrolled in the autopsy and brain donation program of the Joseph and Kathleen Price Bryan ADRC as previously described (Hulette et al., 1998). All subjects gave informed consent. All protocols were approved by the Duke University’s IRB. Neuropathological evaluation was performed following published guidelines (Cairns et al., 2007; Hyman et al., 2012; McKeith et al., 2017; Nelson et al., 2019). Postmortem brain tissues were homogenized at 10% (w/v) in lysis buffer (10 mM Tris pH 7.4, 150 mM NaCl, 0.5% Nonidet P-40, 0.5% deoxycholate, 5 mM EDTA) with mini Beads Beater or hand-held homogenizer. The brain homogenates were further subjected to centrifugation at 1,000 x g for 3 min at 4°C and the supernatants were collected and stored at -80°C for RT-QuIC assays as seeds or for other analyses such as Western blotting.

### Demographic Characteristics and Neuropathological Diagnosis of Clinical Patients

Alzheimer’s disease (AD) patients, non-AD subjects, PiD, PSP, and CBD rare tauopathy patients are as the following: patient ages (in years ± standard deviation) are 71.9 ± 10.1, 72.4 ± 18.3, 74.2 ± 7.0, 77.9 ± 9.7, and 70.3 ± 10.8 for AD, non-AD control, PiD, PSP and CBD patients, respectively. In the same order of these groups, number of male (%) are 11 (61.1%), 12 (63.2%), 0 (0%), 5 (50%) and 3 (33.3%) respectively, and PMI (Post-Mortem Interval; in hours) are 11.2 ± 10.4, 12.0 ± 7.5, 10.2 ± 5.4, 18.9 ± 15.9, and 16.3 ± 12.7, respectively. For the control cohorts, neuropathological diagnosis include mild amyloid angiopathy, mild or moderate atherosclerosis, infarct or microscopic infarcts, microhemorrorhages, metabolic astrocytosis, argyrophilic grain disease. For most of the control subjects, Aβ-plaque scores A0 or A1, the Braak NFT stage scores B0 or B1, and the CERAD neuritic plaque scores C0 or C1. For AD cohorts, most subjects have Aβ-plaque scores A2 or A3, the Braak NFT stage scores B2 or B3, and the CERAD neuritic plaque scores C2 or C3. Some AD cohorts have comorbid conditions of LBD. Rare tauopathies subjects generally have low Thal phases (0–1), indicating no amyloid plaques or plaques limited to neocortex.

### Recombinant Tau Fragment 4RCF and Full Length Human Tau Isoforms Expression and Purification

Plasmids encoding human tau isoforms or truncation mutant 4RCF were transformed into BL21-DE3 E. coli cells. Overnight starter cultures of BL21-DE3 E. coli cells transformed with recombinant tau plasmids were inoculated into multi-liter LB broth at 1:50 dilution and 100 mg/mL ampicillin. Cultures were incubated at 37°C, shaking until OD_600_ reached between 0.5 and 0.6. Tau expression was induced using 1 mM IPTG and continued to grow for an additional 4 h. BL21- DE3 cells containing expressed tau were pelleted and resuspended in 50 mM NaH_2_PO_4_, pH 8.0 and 300 mM NaCl (sonication lysis buffer) at a concentration of 20 mL/L of culture preparation and sonicated at 60% power in ten 30-s intervals over 10 min. An extra boiling step at 90°C for 20 min was added for all full-length tau isoforms before centrifugation (Barghorn et al., 2005). Cell lysates were centrifuged and supernatant containing the protein was applied to Ni-NTA column equilibrated with sonication lysis buffer. The columns were washed with 30-50 times of bed volumes of column buffer followed by washing buffer (50 mM NaH_2_PO_4_, pH 8, 300 mM NaCl, and 20 mM imidazole). Recombinant protein was then eluted using elution buffer (50 mM NaH_2_PO_4_, pH 8, 300 mM NaCl, and 200 mM imidazole). Fractions were tested for protein concentration using 5 μL of protein sample mixed with 10 μL Coommassie Protein Assay reagent (Thermo Scientific). Pooled fractions were concentrated to 4 mL using 10 kD molecular weight cut-off spin columns (Millipore) and filtered using 0.22 μm low-binding Durapore PVDF membrane filters (Millipore). Tau protein was further purified by FPLC using size exclusion Superdex75 and Superdex200 columns (GE Healthcare) in 1X PNE buffer (25 mM PIPES, 150 mM NaCl and 1 mM EDTA at pH 7.0). Purified tau isoforms or tau fragment were evaluated by SDS-PAGE for purity and quantified by BCA protein assays.

### Extraction of Paired Helical Filament-Tau From Alzheimer’s Disease Brains

Paired Helical Filament (PHF)-tau purification follows established protocols (Goedert et al., 1992; Lee et al., 1999; Guo et al., 2016). Briefly, brain tissues from sporadic AD patients with abundant tau pathology qualified for AD were used in this study. All cases used were histologically confirmed. For each purification, 10–12 g of frontal cortical gray matter was homogenized using a homogenizer in 9:1 (v/w) of high-salt buffer (10 mM Tris-HCl, pH 7.4, 0.8 M NaCl, 1 mM EDTA, and 2 mM dithiothreitol, with protease inhibitor cocktail, with 0.1% Sarkosyl and 10% sucrose added and centrifuged at 10,000 g for 10 min at 4°C). Pellets were reextracted once or twice using the same buffer conditions as the starting materials, and the supernatants from all two to three initial extractions were filtered and pooled. Additional Sarkosyl was added to the pooled low-speed supernatant to reach 1%. After 1-h nutation at room temperature, samples were centrifuged again at 300,000 g for 60 min at 4°C. The resulted 1% Sarkosyl-insoluble pellets, which contain pathological tau (PHF-tau), were washed once in PBS and then resuspended in PBS (100 μl/g gray matter) by passing through 27-G 0.5-in. needles. The resuspended Sarkosyl-insoluble pellets were further purified by a brief sonication (20 pulses at 0.5 s/pulse) followed by centrifugation at 100,000 *g* for 30 min at 4°C, whereby the majority of protein contaminants were partitioned into the supernatant, with 60–70% of tau remaining in the pellet fraction. The pellets were resuspended in PBS at one fifth to one half of the pre-centrifugation volume, sonicated with 20–60 short pulses (0.5 s/pulse), and spun at 10,000 g for 30 min at 4°C to remove large debris. The final supernatants, which contained enriched AD PHFs, were used in the study and referred to as PHF-tau or AD-tau. The final supernatant fraction was further analyzed by SDS-PAGE, Western blotting, BCA protein assays, and mass spectrometry.

### Thioflavin-T Fluorescence Aggregation Kinetic Analysis

Fluorescence experiments were performed using a SpectraMax M5 plate reader (Molecular Devices, Sunnyvale, CA). All kinetic reads were taken at 37°C in non-binding all black clear bottom Greiner 96-well plates covered with optically clear films and stirred for 10 s prior to each reading. ThT fluorescence was measured at 444 nm and 491 nm as excitation and emission wavelengths. Each kinetic assay consisted of final concentrations of 30 μM tau protein, 60 μg/ml heparin, and 10 μM ThT. The amount of time required to reach half maximum ThT intensity (t_1/2_) and inhibition constant (IC_50_) values for dose response curves were estimated by multiparameter logistic non-linear regression analysis. The transition from the lag-phase to the growth phase was estimated when the first measurable ThT fluorescence/time (slope) value exceeded ≥5 fold of the previous measured slope (i.e., where the quantum leap in ThT RFU is first noticeable).

### Sarkosyl-Insoluble Tau Pelleting Assay

Purified recombinant tau (10 μM) and heparin (100 μg/ml) were incubated at 37°C for 48 h in 30 mM Tris-HCl, pH 7.5, containing 20 mM DTT. Aggregated tau was assayed on the basis of 1% Sarkosyl insolubility as described below. Aliquots (10 μl) of assembly mixtures were removed and added to 50 μl of 30 mM Tris-HCl, pH 7.5, containing 1% Sarkosyl, and the mixture was left for 30 min. The mixture was then spun at 150,000 × *g* for 20 min. The supernatant (Sarkosyl-soluble tau) was removed, and the pellet (Sarkosyl-insoluble tau) was resuspended in 20 μl of SDS sample buffer containing 5% 2-mercaptoethanol and subjected to SDS-PAGE. Aggregated tau isoforms were visualized after staining of the gel with Coomassie Brilliant Blue.

### Transmission Electron Microscopy Analysis

Transmission electron microscopy (TEM) images were collected as previously described (Velander et al., 2016; Wu et al., 2017). Briefly, 30 μM human tau isoforms were incubated in 20 mM Tris-HCl, pH 7.4 for 10–12 days at 37°C. Prior to imaging, 2 μL of sample were blotted on a 200 mesh formvar-carbon coated grid for 5 min, and stained with uranyl acetate (1%) for 1 min. Both sample and stain solutions were wicked dry (sample dried before addition of stain) by filter paper. Qualitative assessments of the amount of fibrils or oligomers observed were made by taking representative images following a careful survey of each grid. At least 15–20 locations of each grid were observed. TEM was performed on a JEOL-1400 transmission electron microscope (JOEL United States, Inc., Peabody, MA) operated at 120 kV.

### Real-Time Quaking-Induced Conversion Analysis

Tau RT-QuIC assays were conducted as previously described with minor modifications (Orru et al., 2017; Saijo et al., 2017; Kraus et al., 2019; Wang et al., 2019). In brief, each recombinant tau isoform was thawed at room temperature and filtered with a 100 kDa spin column filter (Millipore) to remove unwanted tau aggregates. RT-QuIC reaction mix was composed of 10 mM HEPES, 400 mM NaCl, and 10 μM thioflavin T (ThT) and was filtered through a 0.22 μm filter before use. Each tau isoform was slowly and gently added into the RT-QuIC reaction mix at a final concentration of 12 μM. A 98 μl of RT-QuIC reaction mix containing tau isoform was loaded into each well of a 96-well plate (Nunc). The 10% brain homogenates were diluted at 1:200 in a dilution buffer containing 10 mM HEPES and 1.7% 1 × N2 supplement (Gibco) in 1x PBS and centrifuged at 2,000 *g* for 2 min at 4°C. A 2 μl of diluted brain homogenate was added to the 98 μl of RT-QuIC reaction mix in each well with or without 800 μm silica beads (OPS Diagnostics, Lebanon, NJ). The plate was then sealed with a plate sealing tape (Nalgene Nunc International) and incubated at 37°C in a BMG FLUOstar Omega plate reader (BMG Labtech, Cary, NC) with cycles of 1 min shaking (500 rpm orbital) and 1 min rest throughout the incubation time. ThT fluorescence measurements were monitored every 45 min using 450 ± 10 nm excitation and 480 ± 10 nm emission by bottom reading. Four replicate reactions were prepared at the same dilution for each individual sample. The average fluorescence values per sample were calculated using fluorescence values from all four replicate wells and are shown as one trace. The seeding activity was considered to be tau positive if the ThT fluorescence signal exceeded a threshold reading. The threshold was defined as the average of mean baseline readings for all the negative controls plus four times the standard deviation of those readings.

### Western Blotting Analysis

10% brain homogenates were resuspended in 1x Laemmli sample buffer in sample preparation. Standard procedures were followed as previously described (Wu et al., 2021). Briefly, proteins were transferred to PVDF membranes. The membranes were blocked with 5% non-fat dry milk in PBS-T buffer (0.1% Tween-20 in PBS) for 1 h followed by overnight incubation at 4°C with primary antibody. For human tau isoform detection from postmortem AD brains, anti-tau mAb (HT7; ThermoFisher Scientific, Waltham, MA) was used. After three washes with PBS-T buffer, the membrane was incubated with secondary antibody conjugated with HRP for 1 h at room temperature. The membranes were incubated with Amersham ECL reagents (GE Life Sciences) for signal development after PBS-T and PBS washes.

### Mass Spectrometric Analysis

Paired helical filament (PHF)-tau extracted from neuropathologically diagnosed AD patient brain cortex tissues were processed using S-Trap protocols as detailed by the manufacturer (ProtiFi) and described elsewhere (Gustin et al., 2022). Peptides (10 μl) were injected onto an Easy-nLC 1200 UPLC equipped with a nanospray C18 column (ES801A). The data acquisition and UPLC method lasted for 126 minutes. The mass spectrometer (ThermoFisher Lumos) was operated with an ion spray voltage of 3.0 kV and the ion transfer tube was maintained at 275 degree Celsius. MS data was collected in profile mode while tandem MS data was collected in centroid mode. Raw data was converted to mascot generic format using Proteome Discoverer 2.2 and then searched using Mascot server 2.6.2. The database used was Homo sapiens combined with the Proteome Discoverer contaminants database along with the reversed decoy. Full detailed are provided in the Supplementary Material.

### Statistical Analysis

All data are presented as the mean ± S.E.M and the differences were analyzed with unpaired Student’s *t*-test as implemented within GraphPad Prism software (version 6.0). *p* < 0.05 were considered significant. Tau RT-QuIC data were plotted and analyzed using GraphPad Prism as well.

## Results

### Recombinant Full-Length Tau Isoforms and Engineered Tau Fragment 4RCF Are Capable of Forming Protein Fibrils

All tau isoforms and truncation mutant were purified to homogeneity (>90% purity) by Ni-NTA affinity chromatography and additional steps of size exclusion chromatography. Full-size tau isoforms (Figure 1A) went through an additional boiling step for enhanced purity (> 95%; Figure 1B) (Barghorn et al., 2005). Recombinant human tau isoforms were capable of generating circular oligomers (data not shown) as well as mature fibrils that can be visualized by transmission electron microscopy (TEM; Figure 1C). We noticed that it takes a significantly longer time period (typically 10–12 days under typical incubation conditions) to form tau fibrils, in marked contrast to other more amyloidogenic proteins such as amylin, for which it takes only 2–3 days to form mature fibrils under similar buffer conditions (Velander et al., 2016; Wu et al., 2017).

**FIGURE 3:**
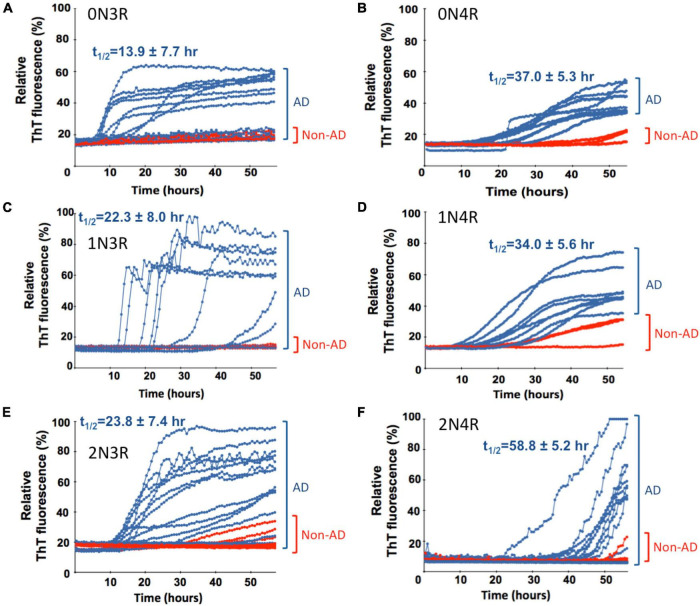
Real-time quaking-induced conversion analyses (RT-QuIC) analysis of seeding activity of misfolded tau from autopsy brain tissues of post-mortem Alzheimer’s disease (AD) and non-AD cadavers in the presence of different recombinant tau isoform (labeled) as a substrate. Tau seeding activity of neuropathologically confirmed AD brains was examined by RT-QuIC assays in the presence of six different individual recombinant full-length tau isoforms. For all panels, seeding activities with AD brain homogenate are shown in blue traces and red traces for non-AD cases. Scattered plots, statistical evaluation of seeding activities, and numbers of cases for AD and non-AD control brains are shown in Figure 4. Average t_1/2_ values for the seeding traces of misfolded tau from AD brains with individual tau isoform substrate are indicated and related scattered plots and statistical evaluation are shown in Supplementary Figure 5.

4RCF tau is an engineered, truncated fragment of the longest isoform of tau isoform 2N4R tau, spanning 2N4R amino acid sequence 244-372 (four repeats of 2N4R) and a carboxy-terminal his_6_-tag to facilitate purification (Figure 2A). Two free cysteine residues C291, C322 were mutated to serine to minimized aberrant inter-molecular crosslinking during expression and purification. Recombinant 4RCF was purified to homogeneity (> 90% purity) and was competent to aggregate and generate protein fibrils (Figure 2A). In comparison with full-length 4R-tau isoforms (0N4R, 1N4R, and 2N4R), 4RCF displayed significantly faster aggregation kinetics, reaching ThT fluorescence signal plateau in approximately 1 h (Supplementary Figure 1) versus 40–60 h for full-length 4R-tau isoforms (Figure 3).

**FIGURE 4:**
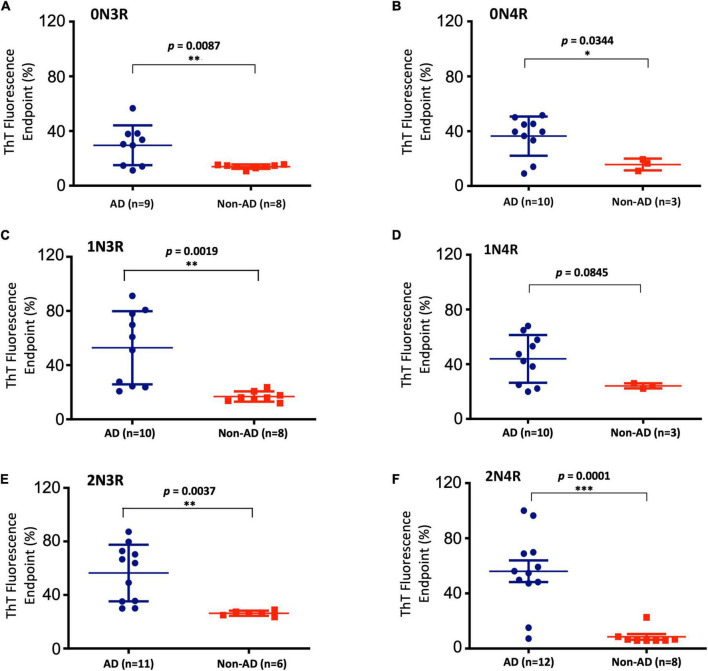
Scattered plot and statistical evaluation of real-time quaking-induced conversion analyses (RT-QuIC) assays of six individual tau isoform (labeled) seeding activities with brain tissues of post-mortem Alzheimer’s disease (AD) and non-AD control subjects as described in Figure 3. GraphPad Prism 6.0 software was used for statistical analyses. Subject numbers in each group are specified. False negative cases are included. *p < 0.05; ^**^p < 0.01; ^***^p < 0.001.

### Verification of Tau Isoforms in Paired Helical Filament-Tau Extracted From Postmortem Alzheimer’s Disease Brains

To verify the existence of multiple human tau isoforms, we purified enriched PHF-tau (AD tau) from postmortem AD brains, following Sarkosyl extraction-based protocols (Lee et al., 1999; Guo et al., 2016). Enriched PHF-tau was clustered between 45 and 70 kD in migration in SDS-PAGE gel (Supplementary Figure 2). These isoforms were confirmed using Western blotting (Supplementary Figure 2) with an anti-tau monoclonal antibody (HT7) that targets the conserved PPGQK sequence (corresponding to residues 159-163 in 2N4R). Our Western blotting analysis showed at least three closely clustered bands (Supplementary Figure 2), in good agreement with PHF-tau preparations described in the literature (Goedert et al., 1992; Guo et al., 2016). Additional TEM characterization of the native tissues showed circular aggregates and fibril species of assembled tau isoforms as reported in the literature (Guo et al., 2016; data not shown). In an effort to identify human tau isoforms, we performed high resolution mass spectrometric analyses of AD brain extracted PHF-tau via a standard bottom-up analysis of released tryptic peptides. The clustered protein bands from SDS-PAGE gel were excised, underwent trypsin-digestion, and resulting fragments were confirmed as human tau isoforms by mass spectrometry (Supplementary Figure 3 and Supplementary Table 1). High-resolution mass spectrometry verified the presence of multiple human tau isoforms by observing the tau isoform fragments (Supplementary Table 1). The results provided unambiguous identification of peptides found in all six isoforms although one cannot provide relative ratios for all six without using labeled standards as there are no prototypic peptides for any individual isoform (Supplementary Figure 3 and Supplementary Table 1).

### 4RCF-Tau-Based Real-Time Quaking-Induced Conversion Assay Selectively Detects Misfolded Tau Seeds in the Alzheimer’s Disease and Related Tauopathies Brains

Real-time quaking-induced conversion analyses (RT-QuIC) assay has been recently used for detection of seeding activities of misfolded proteins, first for prions and then for αSyn and tau *in vitro* (Atarshi et al., 2008; Orru et al., 2017; Kraus et al., 2019). To differentiate misfolded tau seeds in postmortem tauopathy brains from normal controls, an endpoint RT-QuIC-based tau-aggregation seeding activity (tau-ASA) assay was developed using tau fragment 4RCF as the substrate. 4RCF tau has a similar sequence as K18 tau reported in the literature (von Bergen et al., 2001) but with serine mutations at Cys291 and Cys322 (Figure 2A). Such mutations help to avoid protein aggregation during protein expression and purification due to free cysteine mispairing. We examined postmortem brain samples from AD, CBD, PiD, PSP and normal control group by RT-QuIC assays in the presence of 4RCF tau substrate. The concentrations of the substrate and the brain homogenate seeds used for our studies (full-length tau isoform-based RT-QuIC assays included) were determined by our pre-experiments according to recent studies by others (Saijo et al., 2017; Kraus et al., 2019; and Metrick et al., 2020). Specifically, we first conducted the RT-QuIC assays without brain homogenate tau seeds in the presence of different concentrations of recombinant tau substrates but excluded the concentrations that showed self-aggregation during reactions. Then we did seed titrations with selected tau protein concentration to find the best condition showing no or less spontaneous reaction but high sensitivity for our subsequent studies. Tau-ASA, represented by the percentage of tau ThT fluorescence at the endpoint of brain tissues after RT-QuIC assay, indicated significantly higher levels of misfolded tau seeds from tauopathies brains that included 16 cases of AD, 8 cases of CBD, 6 cases of PiD, and 7 cases of PSP (*n* = 37 in total) than those from non-tauopathy controls (*n* = 14) (Figure 2B, *P* < 0.0001 or 0.0004). Corresponding time-dependent kinetic profiles of these RT-QuIC assays with 4RCF substrate are shown in Supplementary Figure 4. This RT-QuIC-based assay, therefore provided a means to differentiate AD and other tauopathies from normal controls. We did not observe any significant differences about seeding activities with 4RCF substrate among samples from 3R-predominant tauopathy (PiD), 4R-predominant tauopathies (PSP, CBD), and 3R/4R mixed tauopathy (AD). Therefore no 3R/4R sample preference was noticed. RT-QuIC ThT signal profiles were heterogenous and overlapping among AD and different tauopathies, albeit average ThT fluorescene for each disease category was significant elevated than the control cases after 50 h of amplification (Supplementary Figure 4).

### Full-Length Tau Isoform-Based Real-Time Quaking-Induced Conversion Analyses Assays Selectively Detect Misfolded Tau Seeds in Alzheimer’s Disease Brains

To further extend our work and to test the *bona fide* substrates in AD brains, we sought to determine whether various recombinant full-length wild-type human tau isoforms can be converted by the tau aggregate seeds from AD brains. We examined autopsied AD and non-AD brain samples from neuropathologically confirmed cadavers (see Materials and Methods). Since there are six tau isoforms present in the human brain (Goedert et al., 1989; Espinoza et al., 2008), we tested how individual isoforms worked as substrates for the RT-QuIC assay of tau aggregation. The tau seeding activities of brain samples from multiple AD and non-AD patients as seeds in the presence of individual six recombinant tau isoforms are shown in Figure 3 and related scattered plots and statistical analyses are shown in Figure 4. Tau-seeding activities were detected starting at approximately 10-30 h and reached a plateau at about 57 h (or >68 h in the case of 2N4R substrate) in AD samples. In contrast, tau-seeding activities in non-AD samples were significantly lower than that in AD samples at ∼57 h (*p* = 0.0087 for 0N3R; *p* = 0.0019 for 1N3R; *p* = 0.0037 for 2N3R; *p* = 0.0344 for 0N4R; *p* = 0.0001 for 2N4R; Figure 4). For 1N4R substrate, tau-seeding activities was marginally higher (*p* = 0.0845) with AD brain homogenates than those with non-AD brain samples. While tau-seeding activities represented by the average ThT fluorescence intensities were significantly higher in AD samples versus non-AD samples, small numbers of false negative cases in the AD sample groups (or end-point overlapping between AD and non-AD in the amplified fluorescent signals) were also observed (Figures 3, 4). Blank controls (tau isoform substrate only and no tissue seeds were added) did not show any seeding activities (data not shown). Based on 10 cases of AD, 2 cases of familiar AD, 2 cases of vascular dementia, and 8 cases of non-AD controls, sensitivity of detection was ranged from 56 to 83.3% and specificity of detection was ranged from 80 to 100% for full-length tau isoform substrates.

### 3R-Tau Isoforms Have Significantly Faster Aggregation Kinetics Than Each of Their 4R-Tau Counterpart

We further analyzed the time course profile of tau RT-QuIC to extract the kinetics information on seeding activities of the misfolded tau by calculating t_1/2_ (time when the fluorescence signal reaches half maximum of each seeding aggregation curve). Seeding aggregation t_1/2_ was estimated to be 13.9 ± 7.7 h, 22.3 ± 8.0 h, and 23.8 ± 7.4 h for 0N3R, 1N3R, and 2N3R substrates and 37.0 ± 5.3 h, 34.0 ± 5.6 h, and 58.8 ± 5.2 h for 0N4R, 1N4R, and 2N4R isoforms, respectively (Figure 3). Full-length 3R-tau substrates exhibited faster seeding kinetics. Pair-wise t_1/2_ comparison of 3R-tau and 4R-tau substrates revealed significantly faster seeding activity kinetics for 3R-tau substrates versus their corresponding 4R-tau counterparts: *p* < 0.001 for 0N3R vs. 0N4R, *p* < 0.05 for 1N3R vs. 1N4R, and *p* < 0.0001 for 2N3R vs. 2N4R (Supplementary Figure 5).

**FIGURE 5:**
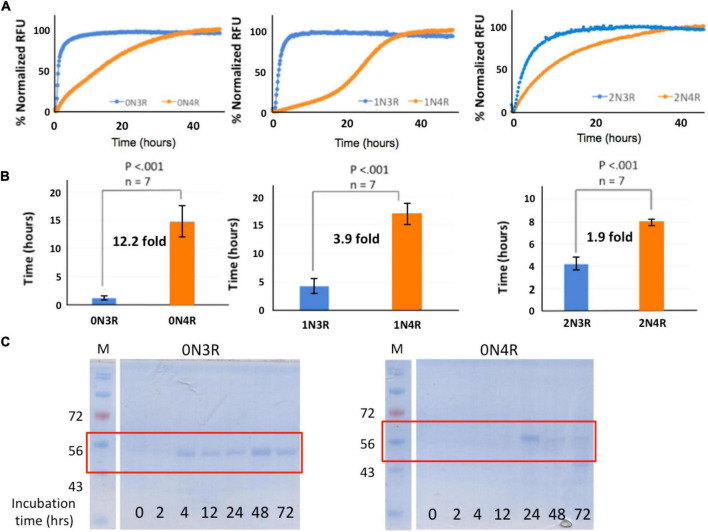
Pair-wise aggregation kinetics comparison of 3R and 4R tau isoforms. **(A, B)** Quantitative analysis of tau isoform aggregation kinetics using ThT fluorescence-based assays. 30 μM of 0N3R or 0N4R (Left Panel), 1N3R or 1N4R (Center Panel), and 2N3R or 2N4R (Right Panel) were incubated in 20 mM Tris pH 7.4 with 0.06 mg/ml of heparin at 37°C for specified time periods. Kinetic fluorescence traces are shown in panel **(A)**. Corresponding t_1/2_ values for each aggregation kinetics curves are shown in panel **(B)**. **(C)** Aggregation kinetics comparison of 0N3R and 0N4R isoforms by Sarkosyl-insoluble tau pelleting assay. 30 μM of 0N3R or 0N4R were incubated in 20 mM Tris pH 7.4 with 0.06 mg/ml of heparin at 37°C for specified time periods. Aggregated tau isoforms (Sarkosyl-insoluble tau) were visualized by SDS-PAGE after Commassie Blue staining.

To further investigate if such isoform-specific kinetics observed in RT-QuIC seeding activities may be recapitulated in “cleaner” biochemical assays (tau isoforms and buffer only and no ingredients from brain tissues), we performed recombinant tau isoform auto-aggregation assays. Consistent with RT-QuIC seeding assays, all the 3R-tau variants auto-aggregated and reached signal plateaus significantly faster (2–20 h) than those for the 4R isoforms (near or after 40 h of incubation) (Figures 5A,B). Pair-wise t_1/2_ comparison of 3R and 4R isoforms mirrored isoform-specific kinetics observed in the RT-QuIC-based postmortem brain tissue seeding assays: 0N3R and 0N4R have t_1/2_s of 1.2 ± 0.4 h and 14.7 ± 3.0 h, respectively, a drastic 12.2-fold change; the 1N3R and 1N4R isoforms have t_1/2_s of 4.3 ± 1.1 h and 17.0 ± 1.6 h, respectively, a 3.9-fold change; and 2N3R and 2N4R have t_1/2_s of 4.2 ± 0.6 h and 8.0 ± 0.3 h, respectively, a 1.9-fold change (Figure 5B). These results demonstrated that microtubule repeat segment 2 (R2)-less 3R-tau isoforms form aggregates much faster compared to the R2-bearing 4R-tau isoforms.

In order to further verify our findings, we performed Sarkosyl-insoluble tau pelleting assays to assess tau assembly kinetics (Taniguchi et al., 2005; Aoyagi et al., 2007). To compare the aggregation kinetics of 0N3R and 0N4R tau isoforms, we examined the lag times of fibrillation in the absence or presence of heparin. No Sarkosyl-insoluble tau was observed with either isoform in the absence of heparin (data not shown). Incubation of tau with heparin resulted in an increase of Sarkosyl-insoluble tau in both isoforms (Figure 5C). Although 0N4R did not exhibit aggregation until 24 h of incubation, 0N3R tau started to aggregate from 4 h of incubation (highlighted in red boxes in Figure 5C) using identical fibril formation conditions, in good agreement with the results from ThT fluorescence kinetic assays (Figures 5A,B). Sarkosyl-insoluble tau pelleting assay was not sensitive enough to detect the difference between 1N3R/1N4R and 2N3R/2N4R pairs, presumably due to their less pronounced kinetic differences (fold of change) than that of 0N3R/0N4R (data not shown).

## Discussion

Applying both concepts and assays derived from prion diseases, we were able to successfully amplify misfolded tau seeds from AD brains using an engineered tau fragment 4RCF, and each of the six individual full-length wild-type tau isoforms as a substrate. There is no reported RT-QuIC study with 4RCF or similar K18 alone as substrate. Previous studies of RT-QuIC seeding assays with other truncated fragments focused on K19 or a longer variant K12 (both are 3R constructs) or mixed fragments (K19 + τ306 or K19 + K18) (Saijo et al., 2017; Kraus et al., 2019; Metrick et al., 2020). Our RT-QuiC assays with 4RCF substrate demonstrated that the four repeating segment alone is capable to amplify misfolded tau seeds from AD and rare tauopathies brains. Separately, to our knowledge, this is the first study reported of selective detection of misfolded tau seeds from AD brains with each of all six full-length tau isoforms as the substrate. In comparison with two recent studies describing ultrasensitive detection of tau aggregate conformer of AD with engineered and significantly shortened tau fragments as substrates (Kraus et al., 2019; Metrick et al., 2020), our study demonstrated such selective detections are also feasible with six full-length human tau isoforms. Non-AD brain samples yielded minimal or significantly lower levels of fluorescent signals for aggregation. Because small numbers of false negative cases in the AD sample groups (or end-point overlapping between AD and non-AD in the amplified fluorescent signals) were observed (Figures 3, 4), we interpret this is likely due to the transition between non-AD and AD is a gradual spectrum (AD disease continuum). Different tau strains can be faithfully amplified *in vitro* from tau isolated from different tauopathy brains and that the amplified tau variants retain their strain-dependent pathogenic characteristics (Xu et al., 2021), our full-length tau isoform-based RT-QuIC assays therefore presumably reflect more faithfully the pathological molecular species of AD brains because full-length tau isoforms are the native forms of tau proteins in human brains (Goedert et al., 1992) and hence offer potentially more accurate diagnosis of AD and other tauopathies for future translational applications. It will be interesting in the future to investigate the structural features of the RT-QuIC end products with substrates of either full-length tau isoforms or truncated tau fragments, and compares them with respective tau fibril structures derived from 3R, 4R, and mixed 3R/4R tauopathies for authenticity. All our tau constructs (full-length isoforms and 4RCF) have a his_6_-tag at C-terminal of each protein to facilitate purification. Because it is far away from the four repeat segments, key region relevant to aggregation, or other structured segment such as N1 and N2, it is highly unlikely the his-tag will affect tau aggregation properties. We have not tested them experimentally, which is a possible limitation.

Results from our ThT fluorescence assays and orthogonal pelleting assays revealed differential aggregation kinetics of the 3R and 4R isoforms. Pair-wise t_1/2_ rate differences (fold changes; Figure 5) may reflect the sequence variations of 0N, 1N and 2N isoforms, specifically the N1 and N2 domains of the tau protein. As the differences between 0N3R/0N4R, 1N3R/1N4R, and 2N3R/2N4R are due to the presence or absence of the microtubule repeat R2 segment, the presence of the R2 repeat, while itself a key aggregation core element, intriguingly slows down the aggregation of the resulting isoforms. Our results are consistent with a recent study reporting that 3R-tau isoforms are more prone to form oligomers than 4R-tau isoforms (Shahpasand-Kroner et al., 2021). However, past work suggested aggregation rate of 3R-tau (or K19) or 4R-tau (or K18) may be redox condition dependent such as the presence or absence of reducing reagent DTT (Schweers et al., 1995; Barghorn and Mandelkow, 2002; Adams et al., 2010). It is currently unknown why the inclusion of a key aggregation R2 element to the 3R tau isoforms yielded corresponding 4R isoforms with slower aggregation kinetics. The fold increase in t_1/2_ was sequentially reduced from 0N3R/0N4R (12.2 fold) to 1N3R/1N4R (3.9 fold), and to 2N3R/2N4R (1.9 fold) in recombinant tau isoform only aggregation systems (Figure 5). Unlike the recombinant tau isoform biochemical systems, there was no decreasing trend in folds with N1/N2 in the RT-QuIC seeding system (2.7-fold for 0N3R/0N4R, 1.5-fold for 1N3R/1N4R, 2.5-fold for 2N3R/2N4R; Figure 3). We interpret that pure recombinant tau isoform aggregation assays are “cleaner” biochemical systems and therefore provide better quantitative molecular insights such as domain contributions to tau protein aggregation. RT-QuIC assays, however, are much more heterogenous and less quantitative. It may complement the pure recombinant tau isoform systems and model better for physiological/pathological states. The physiological and/or pathological significance of these kinetic differences in aggregation remained to be determined. We hypothesize that amino-terminal N1 and N2 segments play some novel roles in tau aggregation partly because the differences in t_1/2_ fold of changes may be assigned to the presence or absence of the N1 and N2 segments of the isoforms evaluated. Roles of N1 and N2 remain to be further investigated and this is an interesting future direction.

Previous studies have demonstrated that all six tau isoforms are present in PHF-tau from AD brains. Most of semi-quantitative estimates of tau amount were based on immunodetection of tau isoforms (Goedert et al., 1992; Lee et al., 1999). Yet detailed quantitative measurements of human tau isoforms within NFTs such as PHF-tau are currently not available. Our mass spectrometric analysis of PHF-tau and recent literature (Barthélemy et al., 2019) demonstrated that it is possible to perform target quantifications in normal and diseased samples if isotope-labeled peptide standards with known quantities are spiked in the testing samples and used as external standards. Such quantification may help to address important yet unresolved questions regarding 3R/4R ratio in normal and diseased CNS samples, and how imbalance of 3R/4R ratios among a combination of tau isoforms affects their aggregation and contributes to AD pathogenesis. Recent development in new technology such as ion mobility-mass spectrometry (Young et al., 2014, 2015) may provide powerful new methods and tools to define protein aggregate intermediates in functionally relevant tau isoform strains.

Tau filaments extracted from AD or other tauopathy brains are heavily decorated with posttranslational modifications (PTMs) (Arakhamia et al., 2020; Wesseling et al., 2020). The PTMs include not only high degree of phosphorylation at dozens of sites, but also other PTMs such as acetylation, ubiquitination, methylation etc. Site-specific PTM has been proposed to play important roles in tau filament formation and AD disease progression (Dujardin et al., 2020; Wesseling et al., 2020). Therefore, it is important to investigate the roles individual PTM site plays in tau aggregation as well as their potential utility for selective detection for tauopathy diagnosis. In this regard, there are significant recent advances in the development of site-specific phosphor-tau biomarkers, such as p-tau181 and p-tau217 for blood- or CSF-based AD diagnosis (Blennow et al., 2015; Janelidze et al., 2020; Thijssen et al., 2021; Leuzy et al., 2022). Nevertheless, *in vitro* assembly of recombinant tau may have its own significant utility because recent study showed that filaments grown from recombinant tau can adopt identical structures as those of AD and chronic traumatic encephalopathy (CTE) (Lövestam et al., 2022). The ease of making AD PHFs and CTE tau filaments *in vitro* with recombinant tau proteins opens new avenues for tauopathies research such as understanding the molecular mechanisms of amyloid formation, small molecule drug screening, filament-specific diagnostic ligand development for positron emission tomography, or special binding agent development to couple the protein degradation machinery aiming to degrade toxic tau filaments inside neurons as potential AD therapy.

Multiple cell-based and *in vivo* mice based tau-seeding experiments provided strong evidence that tau protein has essential characteristics of a prion (Guo and Lee, 2011; Guo et al., 2016; Dujardin et al., 2018). Prion-like propagation of tau aggregates may therefore underlie the disease pathogenesis and progression of neurodegenerative tauopathies. Recent groundbreaking cryo-EM structures of tau filaments from patients with AD, PiD, and CBD further provided atomic evidence of different molecular conformers for several distinct neurodegenerative tauopathies (Fitzpatrick et al., 2017; Falcon et al., 2018; Zhang et al., 2020). Utilizing concepts and techniques such as RT-QuIC originally used in prion disease research, we demonstrated “prion-like” tau isoform seeding activities in diseased AD and related tauopathy brains. For future translational applications, it will be interesting to validate in larger number of disease and control cohorts, to get more accurate estimate of detection selectivity and specificity, and to compare systematically with RT-QuIC assays with various truncated or mixed tau fragments for sensitivity. Important basic science questions also remain to be addressed. For examples, whether tau isoform aggregation plays any roles in disease progression in the context of rapid and slow progressive AD patients? Roles of post-translational modifications such as site-specific phosphorylation and acetylation in tau aggregation? New technological advances will certainly facilitate to address many of such important questions.

## Data Availability Statement

The datasets presented in this study can be found in online repositories. The names of the repository/repositories and accession number(s) can be found in the article/Supplementary Material.

## Author Contributions

LW and ZW performed experiments and acquired and analyzed the data. SL, NG, DD, MM, and FH assisted with the experiments. RH and WR designed and collected and analyzed mass spectrometry data. JL participated in the experiments and provided inputs in experimental design. XZ and SS contributed key reagents and clinical diagnosis data. S-HW verified clinical diagnosis, and collected brain tissues and clinical diagnosis data. GB contributed key reagents and provided inputs in experimental design. W-QZ conceived, designed the experiments, collected brain tissues, acquired and analyzed data, and prepared the manuscript. BX conceived, designed the experiments, analyzed the data, and prepared the manuscript. All authors contributed to the revision and editing of the manuscript.

## Conflict of Interest

The authors declare that the research was conducted in the absence of any commercial or financial relationships that could be construed as a potential conflict of interest.

## Publisher’s Note

All claims expressed in this article are solely those of the authors and do not necessarily represent those of their affiliated organizations, or those of the publisher, the editors and the reviewers. Any product that may be evaluated in this article, or claim that may be made by its manufacturer, is not guaranteed or endorsed by the publisher.

## Abbreviations

AD: Alzheimer’s disease
CBD: Corticobasal degeneration
CNS: Central nervous system
Cryo-EM: Cryo-electron microscopy
CSF: Cerebrospinal fluid
MT: Microtubule
NFT: Neurofibrillary tangle
PBS: Phosphate-buffered saline
PHF: Paired helical filament
PiD: Pick’s disease
PMCA: Protein misfolding cyclic amplification
PSP: Progressive supranuclear palsy
RFU: Relative fluorescence unit
RT-QuIC: Real-time quaking-induced conversion
TEM: Transmission electron microscopy
ThT: Thioflavin T.
