# Supplementary material for: Selective Detection of Misfolded Tau From Postmortem Alzheimer’s Disease Brains
Source: Front Aging Neurosci. 2022 Jul 20;14:945875. doi: 10.3389/fnagi.2022.945875 (PMC9352240; doi:10.3389/fnagi.2022.945875)
Supplement: Supplementary file 1 [file Data_Sheet_1.pdf]

SUPPLEMENTAL MATERIAL

Detailed LC-MS Sample Preparation and Analysis

Samples were processed using S-Trap protocols as detailed by the manufacturer (ProtiFi). Briefly, samples were diluted 2-fold with 2X S-Trap SDS solubilization buffer (10% (w/v) SDS, 50 mM TEAB (pH 7.55). The sample was brought to 4.5 mM DTT by addition of 45 mM DTT and incubated for 1 hour at 37 °C. After cooling to room temperature, the sample was brought to 10 mM iodoacetamide by addition of 100 mM iodoacetamide and incubated in the dark at room temperature for 30 minutes. Unreacted iodoacetamide was quenched by the addition 100 mM DTT (1/9 total volume). One-tenth volume of 12% (v/v) o-phosphoric acid was added followed by 7X S-Trap binding buffer (90% (v/v) methanol, 100 mM TEAB; pH 7.1). Sample was then applied to an S-trap and centrifuged at room temperature for 1 minute at 2000 x g. The S-Trap was washed with S-Trap binding buffer (4X, 150 µL each). Trypsin (1 µg) was then added to the S-Trap in 50 mM TEAB (40 µL, pH 8.0). The S-Trap was incubated overnight at 37 °C. Peptides were recovered and the eluent dried down in a centrifugal vacuum concentrator. The sample was then resuspended in 40 microliters solvent A (2:98 MeCN:H_2O with 0.1% (v/v) formic acid using a sonicating water bath.

Peptides (10 µL) were injected onto an Easy-nLC 1200 UPLC equipped with a nanospray C18 column (ES801A). The data acquisition and UPLC method lasted for 126 minutes. The mass spectrometer (ThermoFisher Lumos) was operated with an ion spray voltage of 3000 and the ion transfer tube was maintained at 275°C. The RF lens was set to 30%. Positive ions with an m/z from 400 to 1500 were detected using the orbitrap operated at 120,000 resolution, an AGC target of 4.0e5 and a maximum injection time of 50 ms. Peaks were chosen for tandem MS if the isotopic distribution resembled that of a peptide with a charge from 2 to 5 and a minimum intensity of 2.0e4. Peaks were dynamically excluded from further tandem MS for 15 seconds (±10 ppm) and apex detection was utilized to perform tandem MS near the most intense portion of the chromatographic peak (expected peak width (FWHM) 15 seconds and desired apex window at 45% peak width). Peaks for tandem MS were isolated using the quadrupole with an isolation window of 1.4 m/z. The HCD collision energy was stepped using steps at 29.1%, 30%, and 30.9%. Fragment ions were detected using an automatic normal scan range beginning at 150 m/z based on the default charge state of 3 using the orbitrap operated at 15000

resolution. The AGC target was 1.0e5 and the maximum injection time was 200 ms. MS data was collected in profile mode while tandem MS data was collected in centroid mode. Raw data was converted to mascot generic format using Proteome Discoverer 2.2 and then searched using Mascot server 2.6.2. The database used was *Homo sapiens* combined with the Proteome Discoverer contaminants database along with the reversed decoy. The enzyme chosen was trypsin with a maximum of two missed cleavages. Fixed modifications: Carbamidomethyl (C); Variable modifications: Deamidation (NQ), Gln->pyro-Glu (N-term Q), Phospho (STY), and Oxidation (M). Peptide Mass Tolerance: 10 ppm; Fragment Mass Tolerance: 0.1 Da.

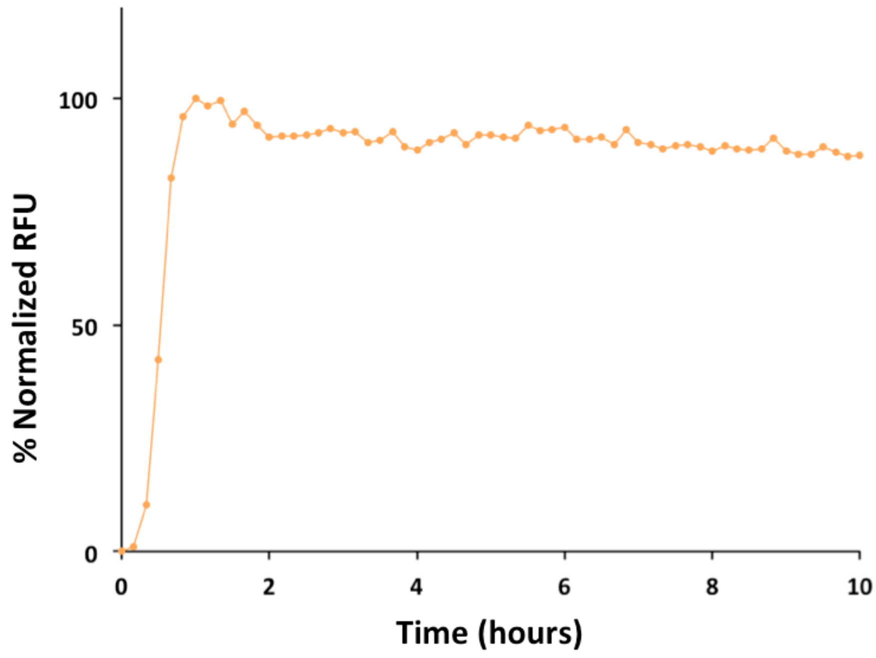

Figure S1. Aggregation kinetics of recombinant 4RCF tau fragment. 30 μM of 4RCF was incubated in 20 mM Tris pH 7.4 with 0.06 mg/ml of heparin at 37 °C for specified time periods. Kinetic fluorescence traces are shown.

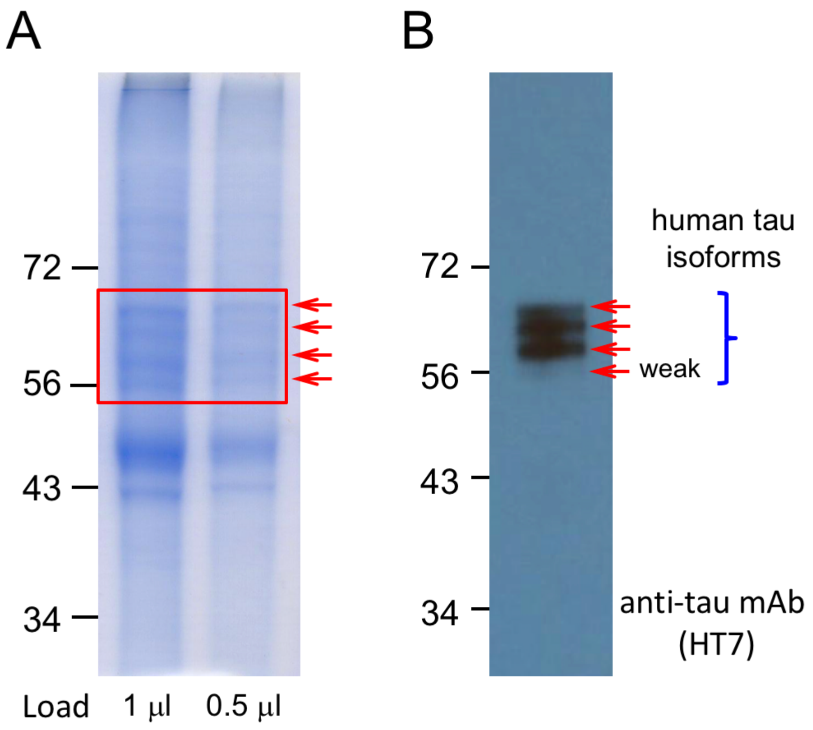

Figure S2. Human tau isoforms extracted from post-mortem AD brains. (A) SDS-PAGE analysis shows authentic human tau isoforms enriched from Sarkosyl extraction and differential ultracentrifugation. (B) Western blot analysis identifies human tau isoforms from AD brain extracted human tau isoform preparations. Anti-tau mAb (HT7; ThermoFisher Scientific, Waltham, MA) was used for human tau isoforms detection.

```
P10636-
(UniProt ID)

0N3R  2  MAEPRQEFEVMEDHAGTYGLGDRKDQGGYTMHQDQEGDTDAGLK----------------  44
1N3R  4  MAEPRQEFEVMEDHAGTYGLGDRKDQGGYTMHQDQEGDTDAGLKESPLQTPTEDGSEEPG  60
0N4R  6  MAEPRQEFEVMEDHAGTYGLGDRKDQGGYTMHQDQEGDTDAGLK----------------  44
2N3R  5  MAEPRQEFEVMEDHAGTYGLGDRKDQGGYTMHQDQEGDTDAGLKESPLQTPTEDGSEEPG  60
1N4R  7  MAEPRQEFEVMEDHAGTYGLGDRKDQGGYTMHQDQEGDTDAGLKESPLQTPTEDGSEEPG  60
2N4R  8  MAEPRQEFEVMEDHAGTYGLGDRKDQGGYTMHQDQEGDTDAGLKESPLQTPTEDGSEEPG  60
         ********************************************

0N3R     ------------------------------------------AEEAGIGDTPSLEDEAAG  62
1N3R     SETSDAKSTPTAE-----------------------------AEEAGIGDTPSLEDEAAG  91
0N4R     ------------------------------------------AEEAGIGDTPSLEDEAAG  62
2N3R     SETSDAKSTPTAEDVTAPLVDEGAPGKQAAAQPHTEIPEGTTAEEAGIGDTPSLEDEAAG  120
1N4R     SETSDAKSTPTAE-----------------------------AEEAGIGDTPSLEDEAAG  91
2N4R     SETSDAKSTPTAEDVTAPLVDEGAPGKQAAAQPHTEIPEGTTAEEAGIGDTPSLEDEAAG  120
                                                   ******************

0N3R     HVTQARMVSKSKDGTGSDDKKAKGADGKTKIATPRGAAPPGQKGQANATRIPAKTPPAPK  122
1N3R     HVTQARMVSKSKDGTGSDDKKAKGADGKTKIATPRGAAPPGQKGQANATRIPAKTPPAPK  151
0N4R     HVTQARMVSKSKDGTGSDDKKAKGADGKTKIATPRGAAPPGQKGQANATRIPAKTPPAPK  122
2N3R     HVTQARMVSKSKDGTGSDDKKAKGADGKTKIATPRGAAPPGQKGQANATRIPAKTPPAPK  180
1N4R     HVTQARMVSKSKDGTGSDDKKAKGADGKTKIATPRGAAPPGQKGQANATRIPAKTPPAPK  151
2N4R     HVTQARMVSKSKDGTGSDDKKAKGADGKTKIATPRGAAPPGQKGQANATRIPAKTPPAPK  180
         ************************************************************

0N3R     TPPSSGEPPKSGDRSGYSSPGSPGTPGSRSRTPSLPTPPTREPKKVAVVRTPPKSPSSAK  182
1N3R     TPPSSGEPPKSGDRSGYSSPGSPGTPGSRSRTPSLPTPPTREPKKVAVVRTPPKSPSSAK  211
0N4R     TPPSSGEPPKSGDRSGYSSPGSPGTPGSRSRTPSLPTPPTREPKKVAVVRTPPKSPSSAK  182
2N3R     TPPSSGEPPKSGDRSGYSSPGSPGTPGSRSRTPSLPTPPTREPKKVAVVRTPPKSPSSAK  240
1N4R     TPPSSGEPPKSGDRSGYSSPGSPGTPGSRSRTPSLPTPPTREPKKVAVVRTPPKSPSSAK  211
2N4R     TPPSSGEPPKSGDRSGYSSPGSPGTPGSRSRTPSLPTPPTREPKKVAVVRTPPKSPSSAK  240
         ************************************************************

0N3R     SRLQTAPVPMPDLKNVKSKIGSTENLKHQPGGGK--------------------------  216
1N3R     SRLQTAPVPMPDLKNVKSKIGSTENLKHQPGGGK--------------------------  245
0N4R     SRLQTAPVPMPDLKNVKSKIGSTENLKHQPGGGKVQIINKKLDLSNVQSKCGSKDNIKHV  242
2N3R     SRLQTAPVPMPDLKNVKSKIGSTENLKHQPGGGK--------------------------  274
1N4R     SRLQTAPVPMPDLKNVKSKIGSTENLKHQPGGGKVQIINKKLDLSNVQSKCGSKDNIKHV  271
2N4R     SRLQTAPVPMPDLKNVKSKIGSTENLKHQPGGGKVQIINKKLDLSNVQSKCGSKDNIKHV  300
         **********************************

0N3R     -----VQIVYKPVDLSKVTSKCGSLGNIHHKPGGGQVEVKSEKLDFKDRVQSKIGSLDNI  271
1N3R     -----VQIVYKPVDLSKVTSKCGSLGNIHHKPGGGQVEVKSEKLDFKDRVQSKIGSLDNI  300
0N4R     PGGGSVQIVYKPVDLSKVTSKCGSLGNIHHKPGGGQVEVKSEKLDFKDRVQSKIGSLDNI  302
2N3R     -----VQIVYKPVDLSKVTSKCGSLGNIHHKPGGGQVEVKSEKLDFKDRVQSKIGSLDNI  329
1N4R     PGGGSVQIVYKPVDLSKVTSKCGSLGNIHHKPGGGQVEVKSEKLDFKDRVQSKIGSLDNI  331
2N4R     PGGGSVQIVYKPVDLSKVTSKCGSLGNIHHKPGGGQVEVKSEKLDFKDRVQSKIGSLDNI  360
             .*******************************************************

0N3R     THVPGGGNKKIETHKLTFRENAKAKTDHGAEIVYKSPVVSGDTSPRHLSNVSSTGSIDMV  331
1N3R     THVPGGGNKKIETHKLTFRENAKAKTDHGAEIVYKSPVVSGDTSPRHLSNVSSTGSIDMV  360
0N4R     THVPGGGNKKIETHKLTFRENAKAKTDHGAEIVYKSPVVSGDTSPRHLSNVSSTGSIDMV  362
2N3R     THVPGGGNKKIETHKLTFRENAKAKTDHGAEIVYKSPVVSGDTSPRHLSNVSSTGSIDMV  389
1N4R     THVPGGGNKKIETHKLTFRENAKAKTDHGAEIVYKSPVVSGDTSPRHLSNVSSTGSIDMV  391
2N4R     THVPGGGNKKIETHKLTFRENAKAKTDHGAEIVYKSPVVSGDTSPRHLSNVSSTGSIDMV  420
         ************************************************************

0N3R     DSPQLATLADEVSASLAKQGL     352
1N3R     DSPQLATLADEVSASLAKQGL     381
0N4R     DSPQLATLADEVSASLAKQGL     383
2N3R     DSPQLATLADEVSASLAKQGL     410
1N4R     DSPQLATLADEVSASLAKQGL     412
2N4R     DSPQLATLADEVSASLAKQGL     441
         *********************
```

Figure S3. Mass spectrometric identification of human tau isoforms and phosphorylation sites of AD tau sample extracted from postmortem AD patient brain tissues. Results are summarized in the format of CLUSTAL O (1.2.4) multiple sequence alignment. Color-coded boxes indicate tryptic peptides identified from tau isoforms. Yellow boxes indicate common tryptic peptides from all six isoforms. See Table S1 for more details on identified tau isoform fragments.

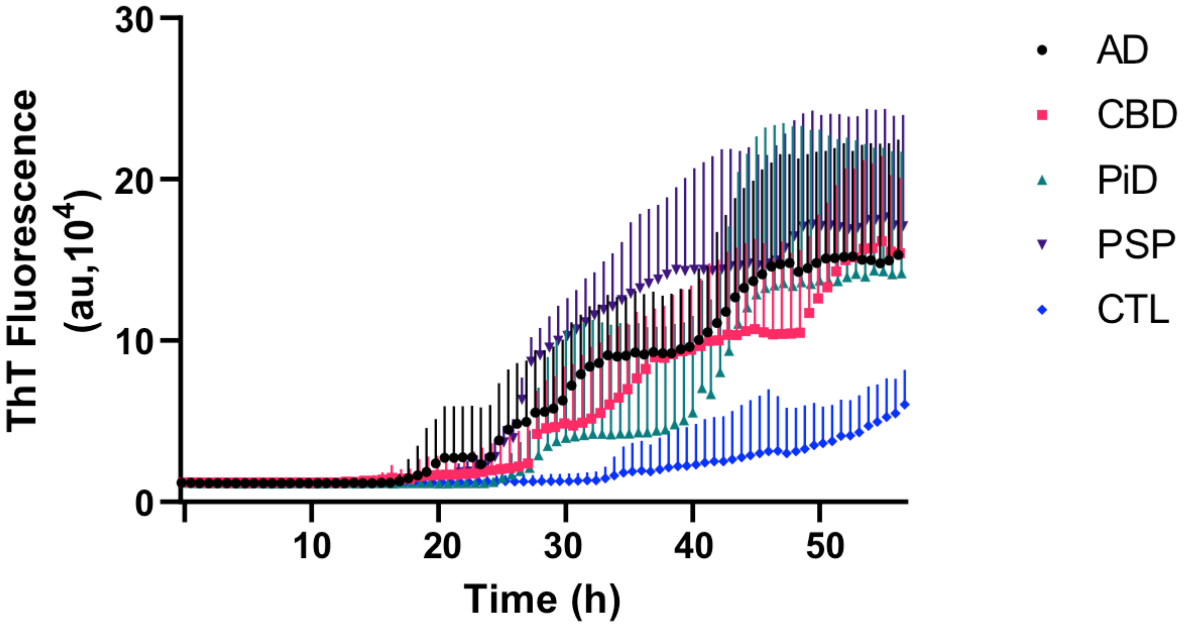

Figure S4. RT-QuIC-based tau-aggregation seeding activity (tau-ASA) assay of brain tissues from tauopathies in the presence of the 4RCF tau substrate. Full kinetic time-courses of tau ThT fluorescence profiles are shown. Corresponding end-point ThT fluorescence plots are shown in Figure 2B. Each tauopathy category has pooled cases and ranges of fluorescence intensity are indicated by vertical lines at each time-point. The brain tissues cases of AD (n=16), CBD (n=8), PiD (n=6), PSP (n=7) and control (CTL, n=14) were examined by RT-QuIC assay.

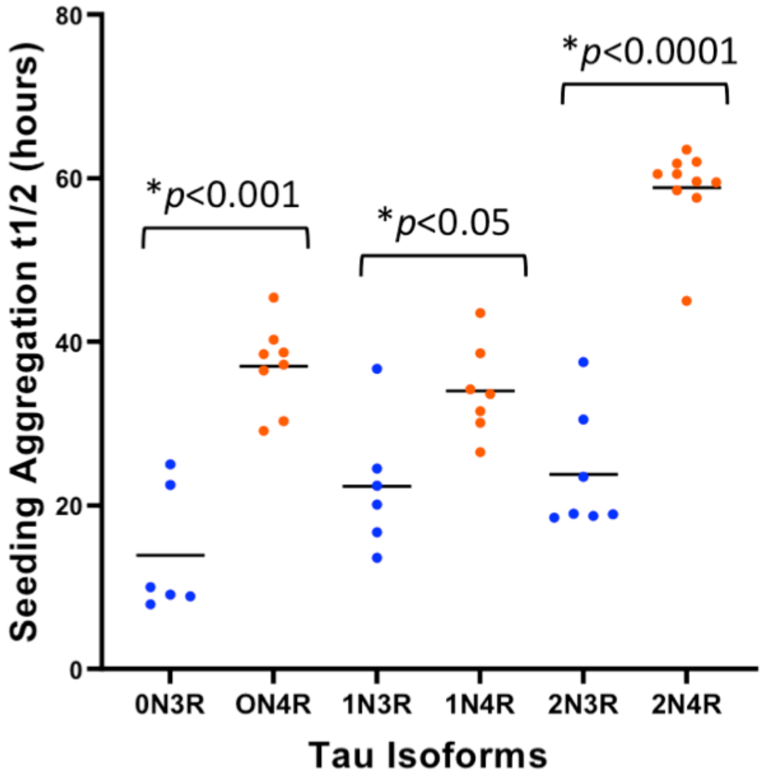

Figure S5. Statistical evaluation of pair-wise comparison of semi-quantitative seeding kinetics ($t_{1/2}$ values) of human tau isoforms with misfolded tau seeds from postmortem AD brains in RT-QuIC analyses as shown in Figure 3. Tau seeding activities of AD brains were examined by RT-QuIC assays with all six individual recombinant tau isoforms. $t_{1/2}$ was defined as time required reaching half maximum or plateaued of ThT fluorescence signals. GraphPad Prism 6.0 software was used for statistical analyses.

Table S1. High Scoring Tryptic Peptides Isolated from Alzheimer's Patient Brain Tissue

Annotated Sequence	Tau Isoforms	Modifications	PSMs	Percolator PEP (Mascot)	Xcorr	Ions Score (Mascot)
AEEAGIGDTPSLEDEAAGHVTQAR	0N3R, 0N4R		4	5.97E-15	6.82	154
KDQGGYTMHQDQEGDTDAGLKAEEAGIGDTPSLEDE- AAGHVTQAR	0N3R, 0N4R		4	8.29E-13	10.76	64
VQIVYKPVDLSK	0N3R, 1N3R, 2N4R		8	1.76E-05	4.54	56
HVPGGGSVQIVYKPVDLSK	0N4R, 1N4R, 2N4R		8	2.71E-08	6.88	58
STPTAEAEEAGIGDTPSLEDEAAGHVTQAR	1N3R, 1N4R		11	1.82E-09	9.18	68
QAAAQPHTEIPEGTTAEEAGIGDTPSLEDEAAGHVTQAR	2N3R, 2N4R		2	1.58E-08	7.38	53
STPTAEDVTAPLVDEGAPGK	2N3R, 2N4R		2	1.59E-08	4.19	78
DQGGYTMHQDQEGDTDAGLK	Common		6	1.17E-08	5.67	64
HLSNVSSTGSIDMVDSPQLATLADEVSASLAK	Common	3x [pS/T]	22	5.57E-08	10.49	79
HLSNVSSTGSIDMVDSPQLATLADEVSASLAK	Common	2x [pS/T]	11	2.98E-08	10.09	87
HLSNVSSTGSIDMVDSPQLATLADEVSASLAK	Common		2	1.28E-11	10.25	104
IGSLDNITHVPGGGNK	Common		4	3.09E-10	6.21	108
IGSTENLKHQPGGGK	Common	Deamidated [N6]	4	2.73E-09	4.37	100
KDQGGYTMHQDQEGDTDAGLK	Common		4	3.90E-12	8.06	106
QEFEVMEDHAGTYGLGDR	Common		14	1.98E-08	6.5	92
QEFEVMEDHAGTYGLGDR	Common	pyro-Glu (N-term)	11	7.75E-09	6.46	67
QEFEVMEDHAGTYGLGDRK	Common	pyro-Glu (N-term)	12	5.60E-08	6.02	71

Quality and Percolator (Sequest and Mascot) q-values were all equal to zero. PSM, Peptide spectrum match; PEP, posterior error probability; Xcorr, Sequest ion score.
